# Berufliche Gratifikationskrisen, Verausgabungsneigung und Burnout bei ukrainischen Anästhesisten und Intensivmedizinern während der SARS-CoV-2-Pandemie

**DOI:** 10.1007/s40664-022-00492-8

**Published:** 2023-01-23

**Authors:** Irina Böckelmann, Igor Zavgorodnii, Olena Litovchenko, Valerij Kapustnyk, Beatrice Thielmann

**Affiliations:** 1 Bereich Arbeitsmedizin, Medizinische Fakultät, Otto-von-Guericke-Universität Magdeburg, Leipziger Straße 44, 39120 Magdeburg, Deutschland; 2 Lehrstuhl für Hygiene und Ökologie No 2, Nationale Medizinische Universität Charkiw, Charkiw, Ukraine; 3 Lehrstuhl für Innere- und Berufskrankheiten, Nationale Medizinische Universität Charkiw, Charkiw, Ukraine

**Keywords:** Effort-Reward-Imbalance, Erschöpfung, COVID-19, Psychische Gesundheit, Prävention, Effort-reward imbalance, Exhaustion, COVID-19, Mental health, Prevention

## Abstract

**Hintergrund:**

Psychische Belastungen von Anästhesisten und Intensivmedizinern sind vielfältig und komplex. Overcommitment (OC) ist durch eine übersteigerte berufliche Verausgabungsneigung gekennzeichnet, die zu psychischen Beanspruchungsfolgen wie z. B. Burnout führen kann. Die Prävalenz von Burnout bei Intensivmedizinern ist international weit gestreut. Ziel der Studie war es, Verausgabungen und Gratifikationen bei ukrainischen Ärzten der Anästhesie und Intensivmedizin während der SARS-CoV-2-Pandemie zu ermitteln und zu analysieren, wie hoch das Burnout-Risiko in dieser Berufsgruppe ist und welche Assoziationen zwischen der intrinsischen Komponente und der extrinsischen Komponente des Modells der beruflichen Gratifikationskrise und dem Burnout bestehen.

**Methodik:**

An der Querschnittsstudie mit der konfirmatorischen Fragestellung im März 2021 nahmen 73 ukrainische intensivmedizinisch tätige Ärzte (47,9 %) und Ärztinnen (52,1 %) teil. Das mittlere Alter lag bei 39,8 ± 10,94 Jahren (Range: 23–78 Jahre). Neben soziodemografischen und berufsbezogenen Daten wurden die berufliche Gratifikation mittels Effort-reward-Imbalance-Fragebogen (ERI) einschließlich der OC-Fragen und das Maslach Burnout Inventory (MBI) erhoben. Die Teilnehmenden der Befragung wurden in Gruppen mit unterschiedlichem OC (< 16 Punkte) und (≥ 16 Punkte) eingestuft und verglichen.

**Ergebnisse:**

In die Gruppe mit OC < 16 Punkte konnten 75 % (55) Teilnehmende eingestuft werden, 18 boten ein erhöhtes OC. Zwischen diesen beiden Gruppen fand sich eine signifikante Differenz im Effort (13,9 ± 4,1 vs. 17,2 ± 3,6 Punkte; *p* = 0,003) und in der ERI-Ratio (0,58 ± 0,2 vs. 0,77 ± 0,2; *p* = 0,006). In der Gruppe mit OC ≥ 16 waren 50 % der Teilnehmenden mit einer hohen emotionalen Erschöpfung (vs. 12,7 % bei OC < 16; *p* = 0,002), aber auch 61,1 % mit einer hohen Leistungsfähigkeit (61,1 % vs. 32,7 %; *p* = 0,005). Insgesamt konnte eine Burnout-Prävalenz von 2,7 % in der Gesamtstichprobe festgestellt werden, wobei die beiden Probanden bei der Gruppe mit OC < 16 vertreten waren. Die höhere emotionale Erschöpfung war mit einer höheren Verausgabungsneigung und Verausgabung (Effort) sowie einer geringeren Belohnung (Reward) assoziiert.

**Diskussion:**

Die Studienergebnisse zeigten hohe emotionale Erschöpfung bei Personen mit hohem Overcommitment. Drei Viertel der Befragten zeigten Burnout-Symptome. Aus diesem Grund sollten Gesundheitsförderungsmaßnahmen und Prävention angeboten werden, um den hohen Belastungen während der Pandemie entgegenzuwirken. Diese sollten Verhältnis- und Verhaltensprävention einschließen.

## Abstract (en)

**Background:**

Mental stress of anesthesiologists and intensive care physicians is varied and complex. Overcommitment (OC) is characterized by an excessive tendency to professional commitment, which can lead to psychological stress and consequently to psychological disorders, such as burnout. The prevalence of burnout among intensive care physicians is internationally widespread. The aim of this study was to determine OC and gratification among Ukrainian anesthesia and intensive care physicians during the SARS-CoV‑2 pandemic and to analyze the risk of burnout in this professional group, and the associations between OC and burnout.

**Methods:**

A total of 73 Ukrainian male (47.9%) and female (52.1%) intensive care physicians participated in the survey. The mean age was 39.8 ± 10.94 years (range 23–78 years). In addition to sociodemographic and occupational data, occupational gratification was collected using the effort-reward imbalance (ERI) questionnaire with additional questions on OC, and the Maslach burnout inventory (MBI). Participants were classified and compared into groups with different OC (< 16 points and ≥ 16 points).

**Results:**

Of the participants 75% (55) could be classified into a group with OC < 16 points, and 18 presented an elevated OC. A significant difference in effort (13.9 ± 4.1 vs. 17.2 ± 3.6 points; *p* = 0.003) and ERI ratio (0.58 ± 0.2 vs. 0.77 ± 0.2; *p* = 0.006) was found between these two groups. In the group with OC ≥ 16 points, 50% of participants reported high emotional exhaustion (vs. 12.7% with OC < 16 points; *p* = 0.002), but also high performance (61.1% vs. 32.7%; *p* = 0.005). Overall, a burnout prevalence of 2.7% was found in the total sample, with both subjects represented in the OC < 16 points group. Higher emotional exhaustion was associated with higher OC, higher effort, and lower reward.

**Discussion:**

The study results showed high emotional exhaustion among subjects with high overcommitment and three quarters of the respondents showed symptoms of burnout. For this reason, health promotion measures and prevention should be offered to counteract the high stresses during the pandemic. These should include relationship and behavioral prevention.

## Hintergrund

Das Burnout-Syndrom, das als ein Zustand mit prozesshafter Entwicklung [15] betrachtet werden kann, wird charakterisiert durch emotionale Erschöpfung, Zynismus/Depersonalisation und reduzierte Leistungsfähigkeit [24]. Burnout führt nicht nur zu Einschränkungen der Leistungsfähigkeit des Betroffenen, sondern auch zu Einschränkungen seiner Lebensqualität und seines Wohlbefindens [1, 15, 28].

Am deutlichsten manifestiert sich das Burnout-Syndrom in Fällen, in denen die berufliche Kommunikation mit emotionaler Intensität und kognitiver Komplexität belastet ist, was typisch für die Tätigkeit von Intensivmedizinern ist.

Bei Burnout entsteht Ungleichgewicht zwischen den Anforderungen und den Ressourcen, welches über einen längeren Zeitraum bestehen bleibt [15]. Es scheint so, dass nicht allein die hohen beruflichen Anforderungen für das Burnout-Risiko verantwortlich gemacht werden können [11]. Möglicherweise sind eine geringere Gratifikation und ein eingeschränkter Handlungsspielraum weitere Ursachen für das Burnout-Risiko [2].

Ärzte^1^ sind bei ihrer beruflichen Tätigkeit körperlichen und psychischen Belastungen ausgesetzt. Die berufliche Tätigkeit von Anästhesisten und Intensivmedizinern ist zusätzlich durch den direkten Kontakt mit schwerstkranken Patienten mit extremen psychischen Belastungen in Kombination mit einer hohen Verantwortung verbunden. Dieser Umstand ist in den allermeisten Fällen mit negativen Emotionen [41], dem Bestehen eigener Ängste um die Patienten auf Intensivstationen und dem Begleiten von Angehörigen [8], der Konfrontation mit den gravierenden Komplikationen und dem Tod [39] verbunden. Das alles kann zu Erschöpfungssymptomen und später zur Entwicklung des beruflichen Burnout-Syndroms beitragen.

Anästhesisten und Intensivmediziner erleben arbeitstäglich komplexe soziale Interaktionen von medizinischer, rechtlicher, ethischer und persönlicher Bedeutung, die oftmals lebenswichtige Entscheidungen in kurzer Zeit einschließen oder die Lebenserhaltung des Patienten während der Behandlungsphasen verantworten [11, 41]. Dazu kommen überlange Arbeitszeiten [11]. Anästhesisten und Intensivmediziner sind koordinierende Bindeglieder zwischen OP-Team, Diagnose- und Behandlungseinheiten. Sie arbeiten in geplanten und unvorhergesehenen Notfallsituationen. Besonders schwierig und verantwortungsvoll ist die präoperative Beurteilung bei Notfällen, wenn das Wissen über den Patienten und das Ausmaß der bestehenden Erkrankung minimal, aber die Verantwortung für ein erfolgreiches Ergebnis maximal ist [41]. Die intraoperative Phase erfordert vom Arzt extreme Belastungen, wie bspw. die minütliche Beurteilung der Situation und des Umfangs des chirurgischen Eingriffs, die Überwachung des physiologischen Zustands des Patienten (visuelle Einschätzung, Monitoring der Vitalparameter und Überwachung der Laborparameter) und die Kontrolle der medikamentösen Behandlung (die Wirksamkeit der Therapie). Das alles bedeutet für den Intensivmediziner eine hohe kognitive Arbeitslast [41].

Im Zusammenhang mit Fortschritten der modernen Medizin und steigenden Zahlen der älteren Patienten hat die Belastung des Personals in der Intensivmedizin zugenommen [15, 41] und nähert sich – u. a. bedingt durch die SARS-CoV-2-Pandemie aufgrund des permanenten Personalmangels – nun dem Limit. Die Pandemie hat erheblichen psychischen und physischen Druck auf die Mitarbeitenden der Intensivstationen erhöht [30].

Krisensituationen sind in der Intensivmedizin die Regel [8]. Jede kritische Situation mit einem Patienten, unabhängig von ihrer Spezifik, ist eine schwere Belastung für den Arzt, die ihn negativ beeinflusst und schließlich zum Burnout führen kann [41].

Viele Studien beschreiben als Folge von hohen Belastungen bei Anästhesisten und Intensivmedizinern psychische und psychosomatische Belastungsreaktionen sowie stressbedingte Erkrankungen, wie z. B. ein Burnout-Risiko [4, 6, 11, 12, 15], Abhängigkeitssyndrome [22], Suizidalität [25], wobei die Vermutung einer überdurchschnittlichen Burnout-Gefährdung von Anästhesisten in Deutschland von Heinke und Koautoren nicht bestätigt wurde [11]. In Österreich kann etwa ein Viertel der Anästhesisten als Burnout-gefährdet angesehen werden [5]. Die Übertragbarkeit der Daten zur Prävalenz des Burnout-Syndroms bei Anästhesisten und Intensivmedizinern aus verschiedenen Ländern auf ein anderes Land sind nur eingeschränkt möglich, da die organisationalen Rahmenbedingungen des Gesundheitssystems sich teilweise massiv unterscheiden. Die Prävalenz liegt daher weltweit zwischen sechs und 47 % [4]. Außerdem sind die eingesetzten Instrumente zur Bestimmung des Burnout-Risikos in verschiedenen Studien nicht einheitlich, was die Vergleichbarkeit der Forschungsdaten erschwert. Zur Prävalenz des Burnout-Risikos unter der Ärzteschaft in Ukraine, u. a. für die Beschäftigten in der Anästhesie und Intensivmedizin, sind kaum Daten vorhanden. Ob die Pandemie die Prävalenz erhöht, wurde noch nicht untersucht und lässt dies nur vermuten. Gerade die laufende SARS-CoV-2-Pandemie ist insbesondere während der ersten Wellen Zeugnis von emotionalen Traumata, posttraumatischen Belastungsstörungen, traumatischen Massenereignissen, moralischen Verletzungen und Burnout bei Personal auf Intensivstationen [27].

Wenn Misserfolgserlebnisse eintreten und die Anerkennung und Wertschätzung von außen (Kollegen, Vorgesetzte, Familienangehörige der Patienten) fehlen, kann es auch zu Krisen beim Einzelnen und im Team auf der Intensivstation im Gesamten kommen. Dies kann die Entwicklung von Burnout-Reaktionen ebenfalls begünstigen [15, 36].

### Theoretisches Erklärungsmodell

Ein theoretisches Modell, das die Zusammenhänge von arbeitsbezogenem psychosozialem Stress und der Gesundheit erforscht, ist das Modell beruflicher Gratifikationskrisen oder Effort-Reward-Imbalance(ERI)-Modell von Siegrist [33, 34]. Im Mittelpunkt dieses Modells steht das Ungleichgewicht (Imbalance) zwischen hoher beruflicher Verausgabung (Effort) und nicht angemessener gewährter Belohnung (Reward). Belohnungen (oder auch Gratifikationen) beinhalten nicht nur die finanzielle Belohnung (Lohn, Gehalt, Prämien), sondern auch Arbeitsplatzsicherheit, beruflichen Aufstieg, Anerkennung und Wertschätzung. Unterschieden wird in dem Modell zwischen extrinsischer (situativer) und intrinsischer (personeller) Komponente [34]. Zur extrinsischen Komponente gehören neben beruflichen Verausgabungen (Efforts) auch die beruflichen Gratifikationen (Rewards). Die intrinsische Verausgabungskomponente ist das Overcommitment (OC) und wird als eine übersteigerte berufliche Verausgabungsneigung betrachtet. Overcommitment ist bedeutsam dafür, ob die berufliche Situation und Merkmale der Arbeitsumwelt als Herausforderung oder als (Fehl‑)Belastung subjektiv erlebt wird und wie sich dies entsprechend auf den Gesundheitszustand und die Leistungsfähigkeit auswirkt [31]. Die Personen, die eine überhöhte Verausgabungsneigung aufweisen, berichten häufiger von Gesundheitsbeeinträchtigungen wie Herz-Kreislauf-Erkrankungen und psychischen Beschwerden [13, 16, 17, 37]. Nach diesem Modell wirkt eine mäßige Verausgabungsneigung und eine gewisse Distanzierungsfähigkeit von der Arbeit und Arbeitsproblemen als eine interne Ressource und gilt damit als gesundheitsprotektiv [34].

Um die Wirkungen von Overcommitment auf die psychische Gesundheit zu untersuchen, soll in der vorliegenden Arbeit der Fragestellung nachgegangen werden, in welchem Verhältnis die Verausgabungen und Gratifikationen bei ukrainischen Ärzten der Anästhesie und Intensivmedizin während der SARS-CoV-2-Pandemie stehen, wie hoch in dieser Berufsgruppe das Burnout-Risiko ist und inwiefern Assoziationen zwischen Verausgabung, Belohnung sowie Overcommitment und dem Burnout gelten. Dabei werden zwei Gruppen mit unterschiedlich ausgeprägter Verausgabungsneigung miteinander verglichen.

## Material und Methoden

Die anonyme Befragung der Ärzte der Intensivmedizin fand im Rahmen eines Kooperationsprojektes „Etablierung der Kriterien für präpathologische Zustände des beruflichen Burnouts bei medizinischem Personal“ (Projektleiter: Prof. I. W. Zavgorodnii) im März 2021 statt. Die Fragebögen wurden in der Papierform vorgelegt. Das positive Votum der Ethikkommission der Nationalen Medizinischen Universität Charkiw vom 17.03.2021 liegt vor.

Im ersten Schritt wurde der Chefarzt des städtischen Prof. K. I. Meshchaninov’s Krankenhauses für Notfallversorgung in Charkiw sowie der Leiter der Abteilung für Notfallmedizin, Anästhesiologie und Intensivmedizin der Nationalen Medizinischen Universität Charkiw schriftlich über das geplante Projekt informiert. Nach Befürwortung des Projektes erfolgte der Versand der Fragebögen an die beiden Einrichtungen. Die Rekrutierung der Probanden wurde durch die Verteilung der Informationen über die geplante Befragung und die Fragebögen durch die Verwaltung vorgenommen, die um eine anonyme freiwillige Teilnahme der Ärzte der Intensivmedizin baten. Die Fragebögen in russischer Sprache (neben dem Ukrainischen die meistgesprochene Sprache in der Ukraine) wurden in Papierform der Ärzteschaft vorgelegt. Von den 130 potenziellen Teilnehmern für diese Befragung antworteten 73. Die Rücklaufquote der Querschnittsstudie entspricht 56 %.

### Stichprobe/Probanden

Die Gesamtstichprobe bestand aus 73 Teilnehmenden aus der Intensivmedizin (35 Männer und 38 Frauen) im Durchschnittsalter von 39,8 ± 10,94 Jahren. Die Altersspanne reicht von 23 bis 78 Jahren.

### Stand der Pandemie in der Ukraine zu dem Zeitpunkt der Befragung

Am 18.03.2021 wurde mit 15.053 COVID-19-Neuinfektionen die höchste Fallzahl seit Ende November 2020 in der Ukraine gemeldet. Der Höhepunkt der vorherigen Welle lag damals bei 16.294 Neuinfektionen innerhalb von 24 h. Die Inzidenz pro 100.000 ist in der Ukraine nicht bekannt. Am 19.03.2021 wurden in der Region Charkiw mit 1085 neu gemeldeten COVID-19-Fällen binnen 24 h ein neuer Negativrekord erreicht. 75 % der COVID-19-Betten waren in dieser Zeit in Kyjiw belegt. Nach einem deutlichen Anstieg der COVID-19-Fallzahlen wurde ein Lockdown veranlasst. Die Zahl der im Zusammenhang mit COVID-19 gemeldeten Todesfälle seit Beginn der Pandemie überschritt mit dem Datum von 22.03.2021 in der Ukraine 30.000 [3].

### Methodik

Im Rahmen der Befragung mit der konfirmatorischen Fragestellung kamen neben den Fragen zu soziodemografischen (Alter, Geschlecht, Familienstand usw.) und berufsbezogenen Daten (Berufsjahre, Tätigkeit usw.) standardisierte, validierte Erhebungsinstrumente ERI-Fragebogen [34] sowie das Maslach Burnout Inventory zur Bestimmung von gesundheitsbezogenen Faktoren bzw. Burnout-Risiko (MBI-GS [23]).

### Effort-Reward-Imbalance-Fragebogen

Der ERI-Fragebogen lag in Langform mit 17 Fragen vor [33]. Er beinhaltet Fragen zur Anstrengung/Verausgabung (Effort) und zur Belohnung (Reward), wobei sich die Fragen zur Belohnung nach Status/Aufstieg, Wertschöpfung/Anerkennung und Arbeitsplatzsicherheit unterteilen. Der Wertebereich für die Skala Effort liegt zwischen 6 und 30 Punkten. Für die Skala Reward können Skalenwerte zwischen 11 und 55 Punkte vorliegen. Die Abschätzung des ERI-bezogenen Gesundheitsrisikos resultiert aus dem berechneten Verhältnis zwischen Effort und Reward (ERI-Ratio) mit der Beachtung des Gewichtungsfaktors [33]. Die ERI-Ratio ermöglicht Aussagen zum Verhältnis von Verausgabung und Belohnung, wobei ein hoher Wert ein hohes Maß an empfundenem Ungleichgewicht von Verausgabung und Belohnung anzeigt. Eine ERI-Ratio > 1 gilt als gesundheitsgefährdend.

Die Fragen zur der beruflichen Verausgabungsneigung (Overcommitment; [33]) können Hinweise auf eine übersteigerte intrinsische Anstrengung bei den Befragten geben. Die Fragen zielen auf Schlafstörungen, Stresslevel und emotionale Belastungen durch die Arbeit ab. Hier liegen die erreichbaren Punktwerte für OC zwischen 6 und 24 Punkten. Je höher der OC-Wert, desto höher die Verausgabungsneigung des Befragten. Als hohes Overcommitment wurden die Werte aus dem 4. Quartil der OC-Verteilung genommen. Ab einem Cut-off-Wert von 16 wird hier von hohem OC gesprochen, d. h. von sehr hoher beruflicher Verausgabungsneigung. Es wurde eine Gruppierung (Gruppe mit einer niedrigen Verausgabungsneigung [OC < 16] und Gruppe mit einer hohen Verausgabungsneigung [OC ≥ 16]) vorgenommen.

### Maslach Burnout Inventory General Survey

Zur Einschätzung des Burnout-Risikos wurde das MBI-GS [23, 29] mit 16 Fragen verwendet. Dabei wurde aus drei erfassten Burnout-Kategorien (emotionale Erschöpfung [EE], Zynismus/Depersonalisation [ZY] und Leistungsfähigkeit [LF]) ein Burnout-Gesamtscore gebildet. Ob ein Burnout-Risiko besteht, wurde anhand der Klassifikation nach Kalimo et al. [14] auf der Grundlage des Gesamtscores ermittelt. Der errechnete Gesamtscore kann drei mögliche Ausprägungen einnehmen: kein Burnout, einige Burnout-Symptome sowie Burnout-Risiko.

### Statistik

Die Auswertung der Daten erfolgte durch die Anwendung des Statistikprogramms Statistical Package of Social Science (SPSS Statistics 28.0., IBM, New York, USA). Zunächst wurden die Daten einer deskriptiven Analyse unterzogen, um Häufigkeiten, Mittelwerte (MW), Standardabweichung (SD), Median sowie Minimum (Min) und Maximum (Max) sowie 95 %-Konfidenzintervall bestimmen zu können. Die Daten auf das Vorliegen der Normalverteilung wurden mit dem Kolmogorov-Smirnov-Test geprüft. Diese lag für die meisten Variablen nicht vor.

Die Prüfung, ob sich die Mittelwerte beider OC-Gruppen statistisch signifikant voneinander unterscheiden, wurde der nichtparametrische Mann-Whitney-U-Test für unabhängige Stichproben angewendet. Mit dem Chi-Quadrat-Test bzw. dem exakten Fisher-Test wurden die Signifikanzen für die Häufigkeitsunterschiede in den Gruppen berechnet. Für alle Berechnungen wurde ein Signifikanzniveau von *p* < 0,05 festgesetzt. Um Assoziationen zwischen den Variablen zu ermitteln, wurde zusätzlich eine Korrelationsanalyse nach Spearman durchgeführt. Die Beurteilung der Effekte von Spearman’s Rho (ρ) erfolgte nach [5] und lautet: < 0,1 kein, ρ = 0,100–0,290 schwacher, ρ = 0,300 bis 0,499 mittlerer und ρ ≥ 0,500 starker Effekt.

## Ergebnisse

### Soziodemographische Daten

Die Geschlechterverteilung in der befragten Gesamtstichprobe (*n* = 73) war vergleichbar (47,9 % Männer und 52,1 % Frauen). Im Durchschnitt waren die intensivmedizinisch tätigen Befragten 39,8 ± 10,94 Jahre alt (Median: 38,0; Range: 23–78 Jahre). Dabei waren 25 % Personen unter 31 Lebensjahren, 50 % unter 38 Lebensjahren und 75 % waren unter 47,5 Jahren.

Von allen Befragten waren 23 (31,5 %) ledig, 34 (46,6 %) verheiratet und 16 (21,9 %) geschieden. Zusätzlich zum Familienstand wurde die Frage gestellt, ob die Teilnehmenden zurzeit in einer Partnerschaft leben. 32 (43,8 %) beantwortete diese Frage mit „nein“, 41 (56,2 %) bejahten das. Von den Ärzten und Ärztinnen, die sich an der Befragung beteiligten, hatten 34 (46,6 %) ein oder mehrere Kinder. 39 der Befragten (53,4 %) hatten „keine Kinder“ angegeben.

Ein geringerer Teil der Befragten 11 (15,1 %) pflegte zu Hause mindestens einen Familienangehörigen.

Das Alter der Probanden beider OC-Gruppen (Gruppe mit einer niedrigen Verausgabungsneigung und Gruppe mit einer hohen Verausgabungsneigung) war nicht signifikant unterschiedlich (Gruppe OC < 16: 39,2 ± 11,16 Jahre vs. Gruppe OC ≥ 16: 41,6 ± 10,33 Jahre; *p* = 0,343).

### Berufsbezogene Daten

Alle 73 Befragten arbeiteten im Bereich der Intensivmedizin, dabei *n* = 6 (8,2 %) in leitender Position. Nur 3 (4,1 %) Ärzte übten zusätzlich regelmäßige Tätigkeiten in der Luftrettung aus.

Die 21 Teilnehmenden (28,8 %) haben primär ein Humanmedizinstudium absolviert. Weitere Ärzte und Ärztinnen hatten vor dem Medizinstudium eine Krankenpfleger-Ausbildung 11 (15,1 %) und 8 (11,0 %) eine Rettungsdienst-Ausbildung (in der Ukraine sog. „Feldscher“) abgeschlossen. 33 (45,2 %) Teilnehmende gaben an, eine sonstige Ausbildung vor dem Medizinstudium gehabt zu haben.

Die Angaben zu den Berufsjahren waren in beiden OC-Gruppen nicht signifikant unterschiedlich (Gruppe OC < 16: 13,5 ± 10,90 Jahre vs. Gruppe OC ≥ 16: 16,4 ± 11,00 Jahre; *p* = 0,189).

### Effort-Reward-Verhältnis und Overcommitment

Die deskriptiven Werte für Overcommitment, die als Grundlage für die Einordnung der Befragten in zwei Gruppen dienten, sind in der Tab. 1 aufgeführt. Die Verausgabungsneigung lag in der Gesamtstichprobe bei 13,4 ± 2,66 Punkten. Die Spannweite für OC dieser Stichprobe lag zwischen 7 und 19 Punkten. Beim Vergleich beider Gruppen mit der unterschiedlichen Verausgabungsneigung wurden nur in der ERI-Skala Effort signifikante Unterschiede festgestellt (13,9 ± 4,08 vs. 17,2 ± 3,62 Punkte). Für die Skala Reward (Belohnung) mit ihren Fragen zum Status/Aufstieg, zur Wertschöpfung/Anerkennung und zur Arbeitsplatzsicherheit wurden keine Differenzen zwischen beiden Gruppen statistisch bestätigt.

**Table 1:** 

ERI	OC < 16 *n* = 55	OC ≥ 16 *n* = 18	Gesamt *n* = 73	*p*_Mann-Whitney_
	MW ± SD [Punkte] Median (Min–Max) 95 % Konfidenzintervall			
Effort (Verausgabung)	13,9 ± 4,08 14 (6–22) 12,79–14,99	17,2 ± 3,62 18 (8–23) 15,42–19,02	14,7 ± 4,21 15 (6–23)	0,003**
Reward (Belohnung)	44,3 ± 5,56 45 (33–55) 42,82–45,83	43,0 ± 6,91 42 (30–52) 39,56–46,44	44,0 ± 5,90 45 (30–55)	0,525
Status/Aufstieg	15,2 ± 2,18 15 (10–20) 14,63–15,81	14,4 ± 2,95 14 (11–19) 12,92–25,86	15,0 ± 2,40 15 (10–20)	0,349
Wertschöpfung/Anerkennung	20,4 ± 3,33 21 (14–25) 19,50–21,30	20,3 ± 3,68 21,5 (15–25) 18,50–22,16	20,4 ± 3,39 21 (14–25)	0,964
Arbeitsplatzsicherheit	8,7 ± 1,49 9 (4–10) 8,31–9,11	8,3 ± 1,97 8 (3–10) 7,30–9,25	8,6 ± 1,61 9 (3–10)	0,525
Effort-Reward-Ratio	0,58 ± 0,191 0,58 (0,23–1,00) 0,533–0,637	0,77 ± 0,241 0,78 (0,28–1,17) 0,646–0,886	0,63 ± 0,218 0,64 (0,23–1,17)	0,006**
Overcommitment (OC, Verausgabungsneigung)	12,3 ± 2,09 13 (7–15) 11,73–12,86	16,7 ± 0,91 16 (16–19) 16,22–17,12	13,4 ± 2,66 13 (7–19)	< 0,001***

**p < 0,01 und ***p < 0,001

Die Effort-Reward-Ratio lag mit 0,77 bei den Befragten mit hohem OC höher als bei den Probanden mit OC < 16. Die Effort-Reward-Ratio war in den beiden untersuchten Gruppen zwar signifikant unterschiedlich (*p* = 0,006), jedoch lag dieser Quotient im Durchschnitt unter 1, d. h. dieses Verhältnis war zwischen Verausgabung und Belohnung nicht gesundheitsgefährdend.

### Burnout-Risiko

Die Befragten mit hoher Verausgabungsneigung haben signifikant höhere emotionale Erschöpfung (*p* < 0,001) und höhere Leistungsfähigkeit (*p* = 0,009; Tab. 2). Die emotionale Erschöpfung der Befragten mit OC ≥ 16 ist im Durschnitt sehr hoch ausgeprägt und liegt mit dem Durchschnittswert von 3,23 minimal über dem Grenzwert von 3,20. Die Leistungsfähigkeit der Befragten der Gruppe mit hoher Verausgabungsneigung (4,85 Punkte) ist noch als *durchschnittlich* zu bewerten. Das Burnout-Risiko ist bei den Probanden mit hohem Overcommitment zwar höher, jedoch nicht statistisch signifikant und liegt im Durchschnitt nach der Klassifikation von Kalimo et al. [14] im Bereich „einige Symptome“.

**Table 2:** 

MBI	OC < 16 *n* = 55	OC ≥ 16 *n* = 18	Gesamt *n* = 73	*p*_Mann-Whitney‑U_
	MW ± SD [Punkte] Median (Min–Max) 95 % Konfidenzintervall			
Emotionale Erschöpfung	1,86 ± 1,223 1,60 (0,0–5,80) 1,528–2,189	3,23 ± 1,533 3,50 (0,40–5,20) 2,471–3,996	2,20 ± 1,426 2 (0–5,80)	< 0,001
Zynismus	1,78 ± 1,231 2,0 (0,0–6,00) 1,445–2,111	1,93 ± 1,314 2,1 (0,20–4,60) 1,280–2,587	1,82 ± 1,245 2 (0–6,00)	0,564
Leistungsfähigkeit	3,35 ± 1,935 2,67 (0,17–6,00) 2,822–3,868	4,85 ± 1,376 5,42 (1,67–6,00) 4,167–5,536	3,72 ± 1,919 4,17 (0,17–6,00)	0,009
MBI-Gesamtscore	2,07 ± 0,839 2,22 (0,18–4,12) 1,846–2,300	2,22 ± 1,017 2,69 (0,62–3,48) 1,712–2,724	2,11 ± 0,881 2,23 (0,18–4,12)	0,300

Zur Einschätzung der Ausprägungsstärke der MBI-Dimensionen siehe 2. Spalte der Tab. 3

**Table 3:** 

MBI-Dimensionen	Ausprägung (Bereich Punkte)	OC < 16 *n* = 55 (%)	OC ≥ 16 *n* = 18 (%)	Gesamt *n* = 73 (%)	*p*_Mann-Whitney‑U_
Emotionale Erschöpfung	Gering (≤ 2,00)	34 (61,8)	4 (22,2)	38 (52,1)	0,002
	Durchschnittlich (2,01–3,19)	14 (25,5)	5 (27,8)	19 (26,0)	
	Hoch (≥ 3,20)	7 (12,7)	9 (50,0)	16 (21,9)	
Zynismus	Gering (≤ 1,00)	18 (32,7)	7 (38,9)	25 (34,2)	0,243
	Durchschnittlich (1,01–2,19)	17 (30,9)	2 (11,1)	19 (26,0)	
	Hoch (≥ 2,20)	20 (36,4)	9 (50,0)	29 (39,7)	
Leistungsfähigkeit	Gering (≤ 4,00)	33 (60,0)	3 (16,7)	36 (49,3)	0,005
	Durchschnittlich (4,01–4,99)	4 (7,3)	4 (22,2)	8 (11,0)	
	Hoch (≥ 5,00)	18 (32,7)	11 (61,1)	29 (39,7)	
Burnout-Risiko	Kein Burnout (0–1,49)	11 (20,0)	6 (33,3)	17 (23,3)	0,394
	Einige Burnout-Symptome (1,5–3,49)	42 (76,4)	12 (66,7)	54 (74,0)	
	Burnout-Risiko (3,5–6,00)	2 (3,6)	0 (0)	2 (2,7)	

*OC* Overcommitment

Die Ergebnisse der Klassifikation der drei MBI-Dimensionen in die drei Ausprägungsgrade („gering“, „durchschnittlich“ und „hoch“) sowie die Ausprägungen des Burnout-Gesamtscores nach Kalimo et al. [14] in drei Klassen („kein Burnout“, „einige Burnout-Symptome“ sowie „Burnout-Risiko“) sind in Tab. 3 dargestellt. Für die MBI-Dimensionen „emotionale Erschöpfung“ und „Leistungsfähigkeit“ wurden signifikant unterschiedliche Verteilungen der Ausprägungsgrade festgestellt (*p* = 0,002 bzw. *p* = 0,005).

Jeder zweite Befragte mit der hohen Verausgabungsneigung (OC ≥ 16) wies hohe Ausprägung der MBI-Dimensionen „emotionale Erschöpfung“ und „Zynismus“ auf. 61,1 % der Probanden dieser Gruppe gaben eine hohe Leistungsfähigkeit an.

Trotz der hohen Verausgabungsneigung der zweiten Gruppe war das Burnout-Risiko und einige Burnout-Symptome bei diesen Befragten mit 66,7 % nicht höher als bei den Probanden mit geringeren OC-Werten (80 %). Berücksichtigt man die zwei Befragten mit dem Burnout-Risiko (Fälle mit Gesamtscore über 3,5), stellt man fest, dass keiner von ihnen eine hohe Verausgabung (OC ≥ 16) angegeben hatte.

Betrachtet man detailliert die vier Probanden mit ERI-Ratio über 1, stellt man fest, dass die vier nach Klassifizierung des Burnout-Risikos nach Kalimo et al. zur Gruppe mit „einigen Symptomen“ einzuordnen waren. Mit dem OC-Medianwert von 17,5 lag die Verausgabungsneigung dieser vier Probanden wesentlich höher als der Medianwert der Gesamtstichprobe (13 Punkte). Diese vier Probanden mit dem MBI-Gesamtscore von 3,17 (Median) blieben noch unter dem Grenzwert von 3,5.

### Assoziationen zwischen der intrinsischen und der extrinsischen Komponente des Modells der beruflichen Gratifikationskrisen und dem Burnout-Risiko

**Figure 1:**
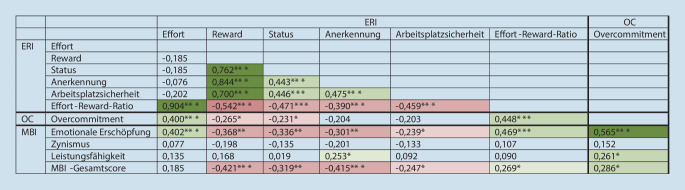

Es bestehen Assoziationen mit einem starken Effekt zwischen Overcommitment und der emotionalen Erschöpfung (ρ = 0,565; *p* < 0,001), der Leistungsfähigkeit (ρ = 0,261; *p* = 0,026) sowie dem Gesamtscore für Burnout-Risiko (ρ = 0,286; *p* = 0,014). Je höher die Verausgabung (ERI Effort) war, desto höher gaben die Befragten an, emotional erschöpft zu sein (ρ = −0,402; *p* < 0,001). Zwischen der Belohnungsskala (ERI Reward) und der emotionalen Erschöpfung besteht eine negative Korrelation (ρ = −0,368; *p* < 0,001). Mit höheren Reward-Werten ist ein geringeres Burnout-Risiko assoziiert. Je höher das Ungleichgewicht zwischen der Verausgabung und Belohnung ist, desto höher ist die emotionale Erschöpfung (ρ = 0,469; *p* < 0,001) und der Gesamtscore für Burnout-Risiko (ρ = 0,269; *p* = 0,021; Abb. 1).

**Figure 2:**
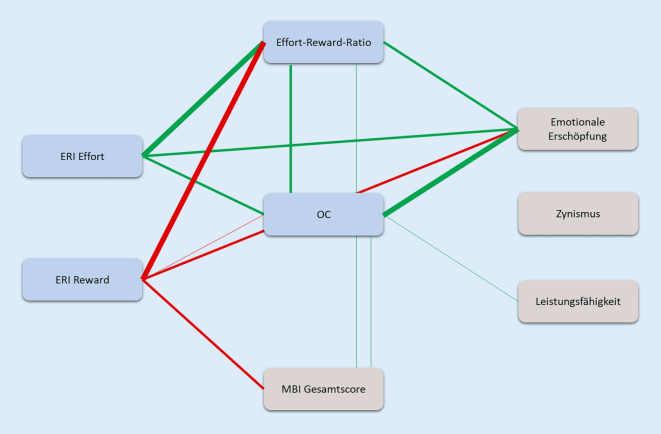

In Abb. 2 sind die Korrelationsverbindungen für die ERI-Hauptskalen, Overcommitment und MBI-Dimensionen zur besseren Veranschaulichung grafisch dargestellt.

## Diskussion

Aufgrund verschiedener Anforderungen und Belastungen, denen Ärzte auf Intensivstationen ausgesetzt sind, stehen sie unter einem erhöhten Risiko, psychische Beanspruchungsreaktionen zu zeigen und das berufliche Burnout-Syndrom zu entwickeln [4, 6, 10–12, 15, 18, 26, 41]. Die gegenwärtige SARS-CoV-2-Pandemie stellt sich auch für Anästhesisten und Intensivmediziner als eine besondere berufliche Herausforderung dar [19].

Die hier vorgestellte Studie untersuchte zuerst, in welchem Verhältnis die Verausgabungen und Gratifikationen bei ukrainischen Ärzten der Anästhesie und Intensivmedizin während der SARS-CoV-2-Pandemie standen und wie hoch die Verausgabungsneigung in dieser Stichprobe ausgeprägt war. Diese werden als eine mögliche Ursache für Burnout betrachtet: Bei Burnout steht oft ein Missverhältnis zwischen den Anforderungen und den Ressourcen über einen längeren Zeitraum [15]. Je höher die Unausgeglichenheit zwischen „Effort“ und „Reward“ ist, desto höher wurde hier das Burnout-Risiko vermutet. Das Verhältnis Effort-Reward-Ratio lag in der untersuchten Stichprobe im Durchschnitt unter 1, was für einen nicht gesundheitsgefährdenden Wert der ukrainischen Ärzte der Anästhesie und Intensivmedizin spricht. Die teilnehmenden Ärzte hatten trotz der SARS-CoV-2-Pandemie eine Ausgeglichenheit zwischen Verausgabung und Gratifikationen. Auch das Overcommitment war in der Gesamtstichprobe im Durchschnitt mit 13,4 Punkten als nicht kritisch zu verzeichnen. Um die Wirkungen von Overcommitment auf die psychische Gesundheit zu untersuchen, wurde dieses als Grundlage für die Gruppeneinteilung gelegt.

In den hier untersuchten Gruppen von ukrainischen Ärzten der Anästhesie und Intensivmedizin mit unterschiedlichem Overcommitment lag das Verhältnis zwischen Verausgabung und Belohnung auch in der Gruppe mit OC ≥ 16 unter dem kritischen Niveau und ist damit auch als gesundheitsungefährlich zu interpretieren. Die Verausgabung und die Belohnung befinden sich in dieser Gruppe in einem guten Verhältnis. Diese Ergebnisse spiegeln sich in den Daten zum Burnout-Risiko wider, die im nächsten Schritt der Studie analysiert wurden. Es wurde zuerst das Burnout-Risiko in dieser Berufsgruppe ermittelt und danach der Frage nachgegangen, inwiefern Assoziationen zwischen Verausgabung, Belohnung sowie Overcommitment und dem Burnout gelten. Im Vergleich der beiden Gruppen mit unterschiedlich ausgeprägter Verausgabungsneigung wurden signifikante Unterschiede in den MBI-Dimensionen „emotionale Erschöpfung“ und „Leistungsfähigkeit“ festgestellt, wobei die Befragten mit hoher Verausgabungsneigung höher und mehr emotional erschöpft waren. Trotz dieses Zustandes der emotionalen Erschöpfung zeigten diese Teilnehmenden aber eine bessere Leistungsfähigkeit. Diese Ausprägungen sorgen dafür, dass der Burnout-Gesamtscore im Durchschnitt noch in dem Bereich „einige Burnout-Symptome“ (nach der Klassifikation nach Kalimo et al. [14]) liegt.

Die internationalen Studien beziffern das Burnout-Risiko in dieser Berufsgruppe mit etwa 25–50 % [4, 11]. Die Arbeitsgruppe um Heinke hat sich in ihrer Studie mit dieser Problematik in Deutschland beschäftigt und stellte fest, dass trotz des Anteils von 40,1 % der 3540 mittels Copenhagen Burnout Inventory (CBI) befragten deutschen Anästhesisten mit einem erhöhten Burnout-Risiko die Burnout-Gefährdung für Anästhesisten in Deutschland nicht größer als für andere Berufsgruppen ist [11]. Diese Autorengruppe zeigte außerdem, dass zwischen Assistenz- und Fachärzten sowie Fach- und Oberärzten keine Unterschiede in dem errechneten Burnout-Score zu finden waren. Diese Ergebnisse waren nicht direkt auf unsere Studie übertragbar, da hier unterschiedliche Instrumente für die Ermittlung des Burnout-Risikos eingesetzt waren. Auch die Differenzierung zwischen Assistenz‑, Fach- und Oberärzten konnte nicht vorgenommen werden, was als Limitation zu betrachten wäre. Bei den Assistenzärzten wäre ein geringeres Burnout-Risiko zu erwarten, da sie nur eine beschränkte Zeit einer Intensivstation zugeteilt sind [15].

Kinzl et al. [15] nutzten den ebenfalls hier angewandten MBI Verfahren und befragten 89 Innsbrucker Anästhesisten. Dabei konnten ähnliche Ausprägungen der MBI-Dimensionen emotionale Erschöpfung bzw. Zynismus/Depersonalisation aufgezeigt werden, allerdings verfügten in der ukrainischen Stichprobe knapp die Hälfte der Probanden (49,3 %) über eine geringe Leistungsfähigkeit (Innsbruck 1/5 der Befragten). 74 % der Probanden boten in der hier vorgestellten Studie einige Burnout-Symptome (vs. Kitzel et al. 25 %) oder hatten ein Burnout-Risiko (2,7 %). Nach dem Modell der Gratifikationskrisen könnte das „Effort“ übersteigende „Reward“ Grund dafür zu sein, dass in dieser Gruppe nur 2,7 % der Teilnehmenden ein erhöhtes Burnout-Risiko aufzeigten. Das ist eine niedrige Prävalenz im Vergleich zu den anderen Daten [11, 15]. Jedoch unter Berücksichtigung der Befragten mit „einigen Symptomen“ ist die Zahl der Burnout-Gefährdeten doch hoch und bedarf Interventionsmaßnahmen, um die Burnout-Gefährdung zu minimieren. Die Vermutung lag nah, dass während der laufenden SARS-CoV-2-Pandemie mit der Zunahme der psychischen Belastungen das Burnout-Risiko steigen wird. Das kann man an dieser Stelle so nicht beurteilen, da die Daten in der Vorpandemiezeit bei ukrainischer Ärzteschaft fehlten.

Die Ergebnisse der Studie belegen eine Assoziation des Burnout-Risikos mit dem „Reward“ (ρ = −0,421), jedoch nicht mit dem „Effort“. Dabei spielt die Anerkennung eine wichtigere Rolle (ρ = −0,415) als der Status (ρ = −0,319) und die Arbeitsplatzsicherheit (ρ = −0,247). Die Anstellungen in den Krankenhäusern in der Ukraine sind meistens unbefristet, so dass in diesem Fall von einer höheren Arbeitsplatzsicherheit per se auszugehen ist.

Starke Assoziationen fanden sich zwischen Overcommitment und der emotionalen Erschöpfung aus dem MBI; bei der anderen MBI-Dimension Leistungsfähigkeit war nur ein schwacher Effekt nachzuweisen. Die MBI-Dimension Zynismus zeigte keine Zusammenhänge zu den ERI-Skalen und OC. Je höher die Verausgabung war, desto höher war die emotionale Erschöpfung bei den Befragten. Auch zeigt sich in dieser Studie eine Assoziation des Overcommitments mit dem MBI-Gesamtscore. Mit der größeren Verausgabungsneigung stieg das Burnout-Risiko. Die Ergebnisse bestätigen Aussagen anderer Studien zum Modell der beruflichen Gratifikationskrisen [7]. Zahlreiche Studien belegen einen Zusammenhang zwischen Overcommitment und Burnout [21, 38], wobei Jobzufriedenheit die Burnout-Symptomatik abmildern konnte [21]. Letztere wurde in der vorliegenden Studie jedoch nicht untersucht. Es bestätigte sich die Annahme von Heinke und Koautoren, dass nicht alleine die hohen beruflichen Anforderungen für das Burnout-Risiko verantwortlich sind [11] und dass die möglichen Ursachen einer geringen Gratifikation zu zuschreiben sind. Eine geringere Gratifikation war in dieser Studie mit einer höheren emotionalen Erschöpfung und einem höheren MBI-Gesamtscore, und folglich mit einem höheren Burnout-Risiko verbunden. Die Ergebnisse der Korrelationsanalyse zeigen nur Zusammenhänge von Effort mit der MBI-Dimension emotionale Erschöpfung.

Es ist bekannt, dass Burnout sich stufen- bzw. prozesshaft entwickelt [20, 23]. In der ukrainischen Stichprobe gaben 50 % der Studienteilnehmende mit erhöhter intrinsischer Verausgabung auch eine hohe emotionale Erschöpfung bei zeitgleich noch guter Leistungsfähigkeit während der laufenden SARS-CoV-2-Pandemie an. Jedoch sollte hier nicht pauschalisiert werden. Es empfiehlt sich bei der Ausarbeitung der Präventionsmaßnahmen und Interventionen, jeden Einzelnen mit seinen Persönlichkeitsmerkmalen, arbeitsbezogenem Verhaltensmuster und Ressourcen („Verhaltensprävention“) zu bewerten, um ggf. den Gefühlen einer chronischen Überforderung oder fehlenden Anerkennung sowie Erfolg- und Machtlosigkeit entgegenzuwirken [20, 23]. Prinzipiell sollten diese Maßnahmen allen Mitarbeitenden angeboten werden, um frühzeitig präventiv das Burnout abzuwehren.

In Deutschland hat sich Burnout zu einem bedeutenden psychosozialen Problem entwickelt, das durch erfolglosen Umgang mit Belastungssituationen und chronischen Stress am Arbeitsplatz verursacht wird. Die Hauptursachen für erlebten Stress in der Arbeit sind auch von den beruflichen Rahmenbedingungen („Verhältnisprävention“) abhängig [15]. An dieser Stelle werden Zeitdruck, häufige Unterbrechungen der Tätigkeit, die fehlenden Ressourcen am Arbeitsplatz sowie eine mangelnde Anerkennung der eigenen Arbeitsleistung durch Vorgesetzte genannt [15]. Folglich sollten diese Verhältnisse in der Primärprävention berücksichtigt werden.

Eine Übersichtsarbeit belegte, dass organisationsgeleitete Maßnahmen mit einer moderaten Reduktion und arztgeleitete Interventionen mit einer geringen Reduktion des Burnout-Scores verbunden waren sowie organisationsgeleitete Interventionen mit einer stärkeren Verbesserung der persönlichen Leistung assoziiert waren [35].

Geeignet zur Reduzierung von Belastungsstörungen erscheinen Angebote zur Resilienz- und Kommunikationsförderung sowie Unterstützungsangebote wie Fall- und Teamsupervisionen, ethische Fallberatungen [30]. Des Weiteren sollte eine feste psychologische/psychotherapeutische Betreuung auf den Intensivstationen etabliert werden [8]. Kollegen und Vorgesetzte als Mentoren und Helfer für schwierige Situationen, die Verständnis und Hilfe auftun, wenn man diese benötigt [40]. Führungskräfte sollten berufliche Belastungen und deren Folgen im Sinne einer Ressourcenschaffung diskutieren und nicht tabuisieren [40], weil diese Belastungen und Beanspruchungen in der Berufsgruppe häufig vorkommen. Ressourcen sollten dabei individuell unter dem Aspekt „Stärken stärken und Schwächen schwächen“ im Rahmen der Verhaltensprävention und Coaching gewonnen werden [9, 15]. Weiterhin kann Verhältnisprävention hohem Beanspruchungsempfinden entgegenwirken (z. B. Handlungs- und Entscheidungsspielraum, Anerkennung durch Vorgesetzte; [15]). Außerdem sind Teilzahlungen für Fitnessclubs zur Verbesserung der Fitness empfehlenswert. Menschen, die Sport treiben, einen gesunden Lebensstil pflegen und die sich gezielt um ihre Physis kümmern, sind deutlich weniger anfällig für Burnout [32, 41].

Zukünftig soll die Forschung darauf abzielen, frühe präpathologische Kriterien für die Entstehung von beruflichem Burnout zu identifizieren und deren Manifestierung zu ermitteln. Für die Praxis ist vorstellbar nach der Etablierung der Kriterien für präpathologische Zustände des beruflichen Burnouts bei medizinischem Personal „Leitfragen“ aus dem Fragebogen zu extrahieren und zu einer Kurzversion des Fragebogens zu erstellen, um die psychologische Korrektur von Burnout-Zuständen frühzeitig vornehmen zu können.

### Limitationen

Die Fallzahlplanung dieser Studie erscheint klein, jedoch orientierte sie sich an Studien gleichen Designs. Aufgrund des kurzen Untersuchungszeitraums ist es möglich, dass nicht alle Ärzte einen Fragebogen erhalten haben. Besonders beanspruchte Ärzte haben möglicherweise auch nicht an der Befragung teilgenommen, weil es ihnen gesundheitlich zu schlecht ging. Somit ist nicht auszuschließen, dass es sich hier um ein Selektionsbias handelt.

Im Rahmen dieser Arbeit wurde keine multiple Regressionsanalyse oder Pfadmodel durchgeführt. Die dargestellten Assoziationen können durch ein Ungleichgewicht von Verausgabung und Belohnung oder andere Variablen beeinflusst sein. Die Möglichkeit eines Confounding wurde hier nicht berücksichtigt. Die Aussagekraft ist begrenzt, da nicht für den Einfluss der anderen Variablen adjustiert wurde.

## Fazit für die Praxis

- Das ausgeprägte Burnout-Risiko ist zwar in dieser Stichprobe gering, allerdings haben 74 % der befragten Intensivmediziner schon einige Burnout-Symptome.
- Die höhere Anerkennung (Reward) wirkt reduzierend auf das Burnout-Risiko.
- Es bestehen positive Korrelationen zwischen der übersteigerten Verausgabungsneigung und der emotionalen Erschöpfung.
- Die im Rahmen dieser Studie gefundenen Assoziationen müssen in weitergehenden Untersuchungen an Stichproben mit der größeren Teilnehmerzahl überprüft werden.
- Maßnahmen der Primärprävention sollten spätestens dann angeboten werden, wenn erste Warnsignale auftreten. Dabei bieten sich Verhältnis- und Verhaltensprävention an und die entsprechende Motivation aller Beteiligten, sich daran partizipativ zu beteiligen.
- Zur Ableitung geeigneter präventiver Maßnahmen ist das Erkennen individueller Risiken und Ressourcen unverzichtbar.
- Weil drei Viertel der befragten Anästhesisten und Intensivmediziner einige Burnout-Symptome oder ein Burnout-Risiko aufweisen, besteht weiterer Bedarf an Forschung und der Suche nach den Gründen für das Entstehen des Burnouts in dieser Berufsgruppe, um die gezielte Interventionsmaßnahmen ableiten zu könnnen.

## Footnotes

1 Aus Gründen der besseren Lesbarkeit wird auf die gleichzeitige Verwendung der Sprachformen männlich, weiblich und divers (m/w/d) verzichtet. Sämtliche Personenbezeichnungen gelten gleichermaßen für alle Geschlechter.
